# The Anti-Myogenic Role of Tetranectin and Its Inhibition by Epigallocatechin-3-Gallate Enhances Myogenesis

**DOI:** 10.3390/cells14151160

**Published:** 2025-07-28

**Authors:** Amar Akash, Jihoe Kim

**Affiliations:** Department of Medical Biotechnology, Yeungnam University, Gyeongsan 38541, Republic of Korea; amarakash@yu.ac.kr

**Keywords:** myogenesis, tetranectin, anti-myogenic factor, epigallocatechin gallate, muscle regeneration

## Abstract

Tetranectin (TN) is a plasminogen-binding protein found in human serum. Although it has been suggested to be closely related to various stem cell differentiation, including myogenesis, the role of TN in muscle development remains unclear. In this study, we identified TN as an anti-myogenic factor during the differentiation of C2C12 satellite cells. The exogenous supplementation of TN inhibited myogenic differentiation, whereas differentiation was significantly enhanced in the TN-depleted medium. Epigallocatechin-3-gallate (EGCG), a catechin abundant in green tea, significantly enhanced myogenic differentiation by reducing TN levels in the medium and downregulating TN gene expression during the differentiation process. These results demonstrate that EGCG promotes myogenesis by inhibiting TN at both the transcriptional and functional levels, highlighting TN as a promising therapeutic target for muscle regeneration disorders.

## 1. Introduction

Tetranectin (TN) is a plasminogen-binding protein found in the blood, produced by various types of cells in multiple tissues [1,2]. Serum levels of TN are significantly reduced in cancer patients, while protein accumulates in the extracellular matrix of tumor tissues, suggesting its potential as a prognostic marker [3,4]. TN facilitates the conversion of plasminogen to plasmin, implicating it in extracellular matrix remodeling associated with cancer development and metastasis [5,6]. Besides cancer, TN is also involved in stem cell differentiation processes, including adipogenesis, osteogenesis, and myogenesis [6,7,8]. It is highly expressed in developing tissues such as the limbs and lungs and is also detected in various body fluids. Notably, TN expression increases during muscle regeneration and muscle cell differentiation, indicating its potential regulatory role [8]. However, its functional role in muscle cell differentiation remains poorly understood.

Epigallocatechin-3-gallate (EGCG) is a polyphenolic compound and the most abundant catechin found in green tea [9]. It has been shown to exert a wide range of beneficial biological effects, including anti-inflammatory, anticancer, and cardioprotective activities [10,11]. Recent studies have suggested that EGCG may enhance skeletal muscle mass and improve physical performance, suggesting its potential as a therapeutic agent for sarcopenia [12]. Although the mechanism underlying these effects remains to be fully elucidated, EGCG has been reported to promote the differentiation of muscle stem cells, thereby enhancing muscle regeneration [13,14].

In this study, we identified TN as an anti-myogenic protein that suppresses the myogenic differentiation of murine C2C12 satellite cells. Furthermore, we demonstrated that EGCG reduces TN proteins in the culture medium and downregulates TN gene expression during the differentiation process. These findings uncover the mechanism by which EGCG promotes myogenic differentiation and suggest that TN is a promising therapeutic target for muscle regeneration disorders.

## 2. Materials and Methods

### 2.1. Cell Culture and Myogenic Differentiation

Murine C2C12 myoblast cells (Korean Cell Line Bank, Seoul, Republic of Korea) were cultured in Dulbecco’s modified Eagle medium (DMEM) containing 10% fetal bovine serum (FBS) and 1% penicillin/streptomycin (Hyclone) at 37 °C in a humidified environment with 5% CO_2_. For the myogenic differentiation, C2C12 cells (3 × 10^4^) were seeded in a 6-well plate and grown in a growth medium (DMEM with 10% FBS) until approximately 90% confluency (Day 0). Differentiation was initiated by switching the media (DMEM with 2% FBS), and this was followed for 6 days with media exchanges every second day. The indicated concentrations of exogenous TN and EGCG were added at differing initiations and with each subsequent media exchange. In another experiment, TN-depleted FBS (TN-del-FBS) was prepared as stated in the previous report [15] and used for the myogenic differentiation of C2C12 cells in DMEM with 2% TN-del-FBS.

### 2.2. Preparation and Activity Assay of Recombinant TN Proteins

Recombinant bovine and mouse TN (rbTN and rmTN, respectively) were prepared as previously described [15]. Briefly, the corresponding TN genes in pET28a(+) were expressed in *E. coli* BL21 grown in an LB medium (500 mL) at 37 °C. Gene expression was induced at OD A600 nm ≈ 0.7 by adding 0.5 mM isopropyl β-d-thiogalactopyranoside (IPTG). After overnight incubation, cells were harvested and lysed by sonication (5:30 s/10 min/40% amplitude) in 20 mL of lysis buffer (50 mM Tris-HC1 pH 8.0, 0.5 M NaCl, 1 mM PMSF, and 150 µg/mL lysozyme). Insoluble parts were collected by centrifugation at 16,000 rpm and solubilized in 10 mL of denaturing buffer (50 mM Tris-HC1 pH 8.0, 0.5 M NaCl, 8 M urea, and 10 mM β-mercaptoethanol). TN was purified by a Ni-NTA affinity chromatography under denaturing conditions, followed by a cyclic on-column refolding procedure at 4 °C. The Ni-NTA column was first washed with five column volumes of buffer A (50 mM Tris-HCl pH 8.0, 0.5 M NaCl, 2 mM CaCl_2_, 2 mM reduced glutathione, and 0.2 mM oxidized glutathione), followed by 1.5 column volumes of buffer B (50 mM Tris-HCl pH 8.0, 0.5 M NaCl, 8 M urea, 2 mM CaCl_2_, and 3 mM reduced glutathione). This refolding cycle was repeated with a 5% stepwise decrease in buffer B by replacing it with buffer A, generating a gradient from 100% to 15% for buffer B. Refolded TN was eluted using an imidazole gradient (50–300 mM), pooled, and imidazole was removed by buffer exchange into 50 mM Tris-HCl (pH 8.0) and 500 mM NaCl using a 10 kDa cut-off Centricon (Satorius, Stonehouse, UK).

TN was prepared in the buffer with 50 mM Tris-HC1, pH 8.0, and 0.5 M NaCl.

The activity of recombinant TN was confirmed using a plasminogen (Plg) activation assay, as described in [16]. Briefly, the assay mixture contained 0.1 µM Plg, 1 µM recombinant TN, and 0.5 mM of the plasmin substrate D-Val-Leu-Lys-p-nitroaniline. The reaction was initiated by adding 0.5 nM of the tissue-type plasminogen activator, and the release of p-nitroaniline was continuously monitored by measuring absorbance at 405 nm.

### 2.3. Determination of Fusion Index and Creatine Kinase Activity

Differentiated cells were stained with Giemsa stain (Sigma Aldrich, St. Louis, MO, USA) following the manufacturer’s protocol, and fusion indices were calculated as previously described [17]. Creatine kinase activities were determined for differentiated cells using a creatine kinase assay kit (BioAssay Systems, Hayward, CA, USA) following the manufacturer’s protocol.

### 2.4. Real-Time Quantitative PCR for Gene Expression Analysis

Total RNA was extracted from cells using the Trizol reagent (Invitrogen, Carlsbad, CA, USA), and cDNA was synthesized using a reverse transcription kit (Applied Biosystems, Foster City, CA, USA), following the manufacturer’s protocol. Gene expressions were analyzed using cDNA, primers specific for indicated marker genes (Supplementary Table S1), and the Power SYBR green mix PCR master mix (Thermo Fisher Scientific, Warrington, UK). Relative gene expressions were determined and normalized with controls, calculating 2 − ∆Ct, where ∆Ct = Ct gene − Ct control. All determinations were averages from at least three triplicates (*n* ≥ 3) with standard deviations.

### 2.5. Western Blot Analysis

Cells were harvested under the indicated conditions, and total cell proteins were prepared by cell lysis using the RIPA buffer, which contained a protease inhibitor cocktail (Thermo Fisher Scientific, Waltham, MA, USA). Proteins were separated by 12% SDS-PAGE and transferred to PVDF membranes that were blocked with 5% skimmed milk in TBST (Tris-buffered saline with 0.1% Tween-20 and incubated with a protein-specific primary antibody (Supplementary Table S2). After overnight incubation at 4 °C, the membrane was incubated with the HRP-conjugated secondary antibody, and signals were obtained using the D plus Pico Chemiluminescent Substrate (Dongin LS, Seoul, Republic of Korea).

Proteins in differentiation media (~10 μg/10 μL) were also analyzed by Western blot using the anti-TN antibody (Supplementary Table S2). Media TN signals were quantified using Image J software version 1.49 and normalized with albumin (~66 kDa), the most abundant serum protein, separated by 12% SDS-PAGE.

### 2.6. Statistical Analysis

The significance of differences was determined by SPSS 22.0 (IBM, Armonk, NY, USA) using the unpaired Student *t*-test and one-way ANOVA. Results are presented as means ± standard errors, and a *p*-value ≤ 0.05 (*) or ≤0.01 (**) was considered statistically significant.

## 3. Results

### 3.1. Upregulation of TN During Myogenic Differentiation and Its Secretion

**Figure 1:**
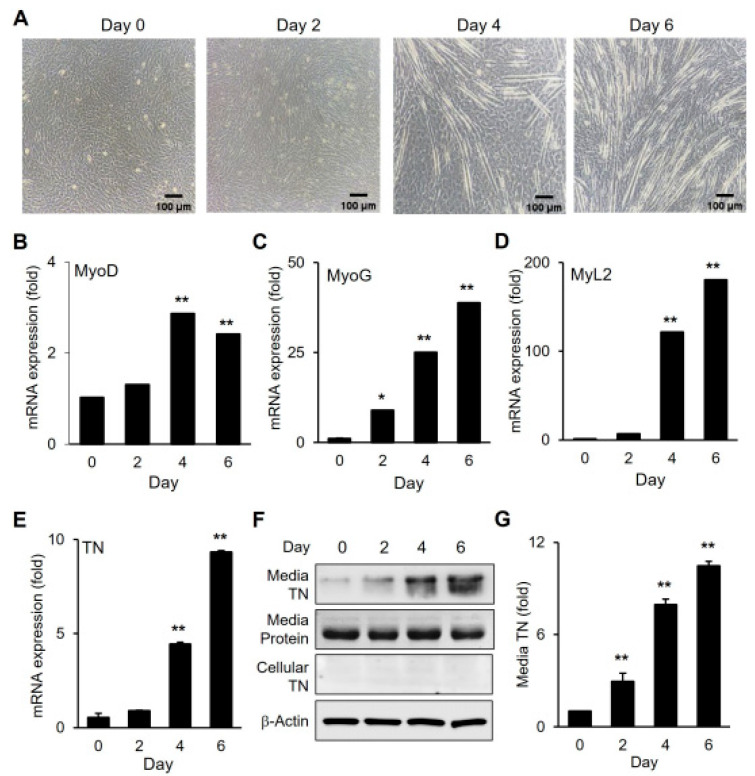
The upregulation of TN and protein secretion. (**A**) Microscopic figures (bright field) on the days of myogenic differentiation of C2C12 cells. (**B**–**E**) mRNA expression of the myogenic marker genes and TN gene. (**F**) Immunoblotting for TN in media and in cells following differentiation. (**G**) Quantitative analysis of (**F**) using ImageJ software. Values from *n* ≥ 3 with standard deviations and *p* values ≤ 0.05 (*) or ≤0.01 (**).

The expression of tetranectin (TN) was examined during the myogenic differentiation of C2C12 satellite cells (Figure 1). As differentiation progressed, myotube formation increased, accompanied by the upregulation of myogenic marker gene expression (Figure 1A–D). TN gene expression was significantly elevated following differentiation, reaching its peak on day 6 of differentiation (Figure 1E). TN was undetectable in total cell lysates, but it was detected in the differentiation medium (Figure 1F,G). Moreover, TN levels in the medium increased in parallel with gene expression, indicating the extracellular secretion of TN during myogenic differentiation.

### 3.2. TN Suppresses Myogenic Differentiation

**Figure 2:**
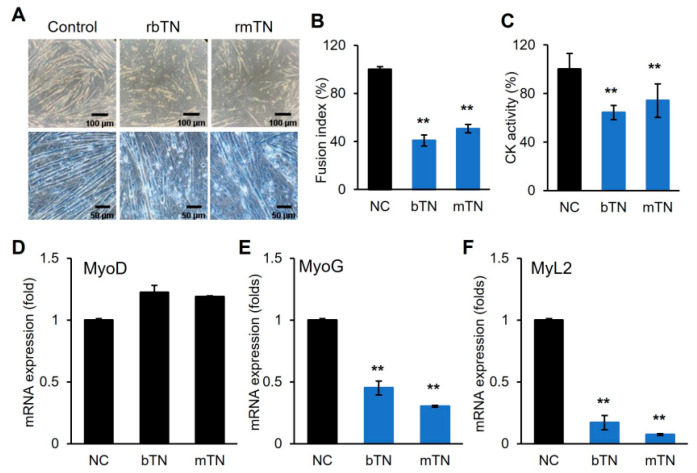
Suppression of myogenic differentiation by exogenous TN supplementation. (**A**) Comparison of microscopic figures on day 6 of differentiation with exogenous TN (2 μM) supplementation (rbTN, recombinant bovine TN; rmTN, recombinant mouse TN). Upper, bright field; lower, Giemsa stain. (**B**,**C**) Comparison of fusion index and creatine kinase activity (CK). (**D**–**F**) mRNA expression of myogenic marker genes. Values from *n* ≥ 3 with standard deviations and *p* values ≤ 0.01 (**).

**Figure 3:**
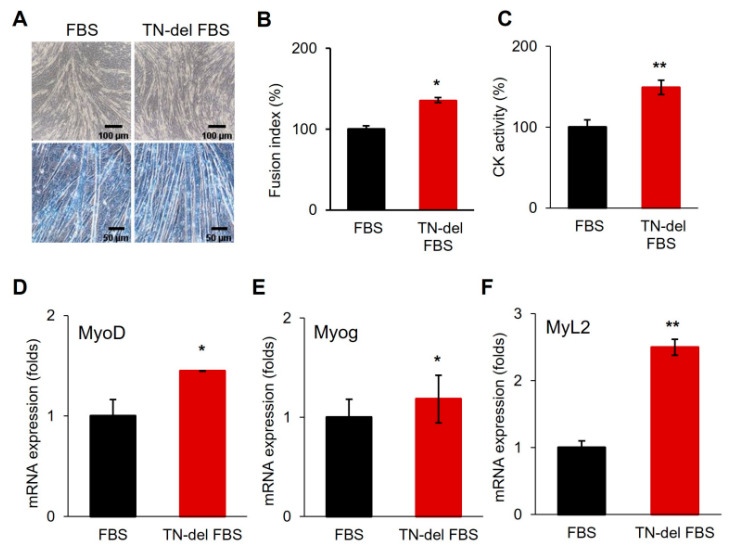
Enhanced myogenic differentiation in TN-depleted media. (**A**) Comparison of microscopic figures on day 6 of differentiation (TN-del-FBS, TN-depleted FBS media). Upper, bright field; lower, Giemsa stain. (**B**,**C**) Comparison of fusion index and creatine kinase activity (CK). (**D**–**F**) mRNA expression of myogenic marker genes. Values from *n* ≥ 3 with standard deviations and *p* values ≤ 0.05 (*) or ≤0.01 (**).

The recombinant TN protein was prepared and supplemented into the differentiation media of C2C12 cells (Figure 2). The exogenous supplementation of TN inhibited the myogenic differentiation, as indicated by significant decreases in myotube formation, the fusion index, and creatine kinase (CK) activity (Figure 2A–C). The myogenic marker genes, MyoG and MyL2, were consistently downregulated, whereas MyoD exhibited insignificant changes (Figure 2D–F). In a separate experiment, a differentiation medium was prepared using TN-depleted FBS (TN-del-FBS) instead of normal FBS and used for the myogenic differentiation of C2C12 cells (Figure 3). Differentiation was enhanced in the TN-del-FBS medium compared to the control medium, as evidenced by significant increases in myotube formation, the fusion index, and CK activity (Figure 3A–C). Consistently, myogenic marker genes were also significantly upregulated (Figure 3D–F). These results indicate that TN acts as an anti-myogenic factor, suppressing the myogenic differentiation of C2C12 cells.

### 3.3. EGCG Promotes Myogenic Differentiation

**Figure 4:**
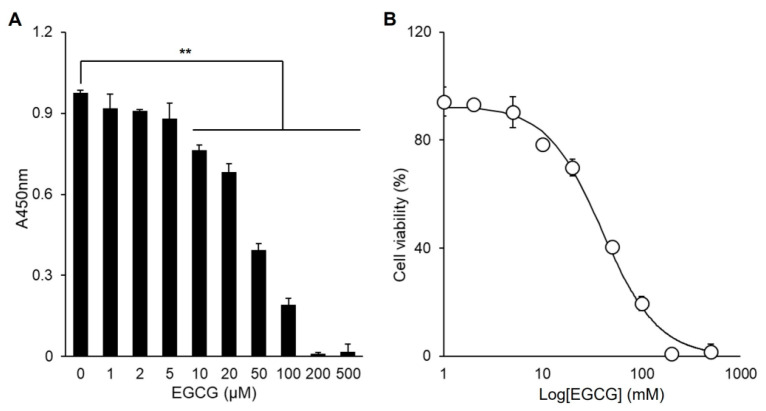
Determination of EGCG cytotoxicity. (**A**) Viable cells at the different EGCG concentrations, as described in the Section 2. (**B**) The determination of IC50 = 40.0 ± 0.3 µM by fitting the data in (**A**) to a logarithmic function (solid line). Values from *n* ≥ 3 with standard deviations and *p* values ≤ 0.01 (**).

**Figure 5:**
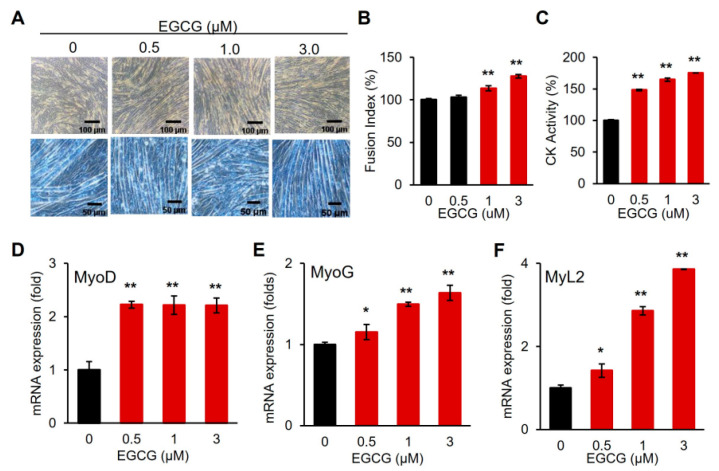
EGCG-promoted myogenic differentiation. (**A**) Comparison of microscopic figures on day 6 of differentiation at the indicated EGCG concentrations. Upper, bright field; lower, Giemsa stain. (**B**,**C**) Comparison of fusion index and creatine kinase activity (CK). (**D**–**F**) mRNA expression of myogenic marker genes. Values from *n* ≥ 3 with standard deviations and *p* values ≤ 0.05 (*) or ≤0.01 (**).

EGCG significantly inhibited the proliferation of C2C12 cells at concentrations ≥ 10 µM, with an IC50 = 40.0 ± 0.3 µM (Figure 4A,B). However, at low concentrations (≤5 µM), EGCG did not significantly affect cell proliferation. Therefore, the effect of EGCG on the myogenic differentiation of C2C12 cells was examined at these low concentrations (Figure 5). EGCG supplementation enhanced differentiation, showing dose-dependent increases in myotube formation, fusion index, and CK activity (Figure 5A–C). Consistently, the expression of myogenic marker genes also increased (Figure 5D–F), indicating that EGCG promotes the myogenic differentiation of C2C12 cells.

### 3.4. EGCG Removes TN from Differentiation Medium and Downregulates TN Gene Expression

**Figure 6:**
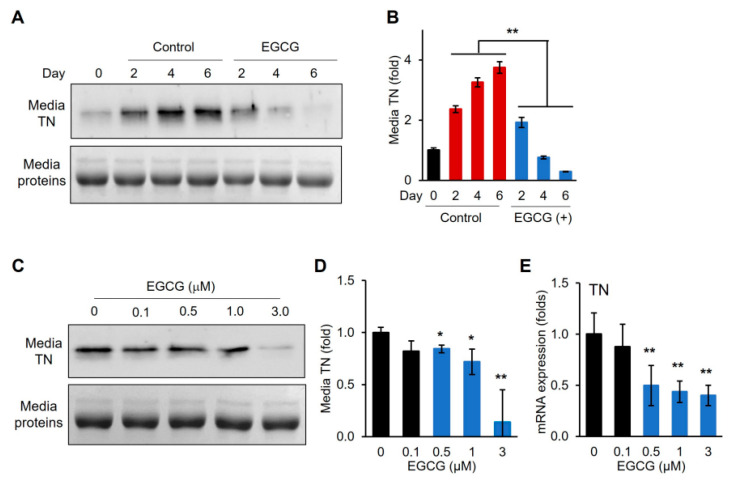
Depletion of TN in media and downregulation of TN gene expression by EGCG. (**A**) Immunoblotting for TN in media following differentiation. (**B**) Quantitative analysis of (**A**) using ImageJ software. (**C**) Immunoblotting for TN in media on day 6 of differentiation at indicated EGCG concentrations. (**D**) Quantitative analysis of (**C**) using Image J software. (**E**) mRNA expression of TN gene. Values from *n* ≥ 3 with standard deviations and *p* values ≤ 0.05 (*) or ≤0.01 (**).

We previously demonstrated that EGCG binds to TN and inhibits its function in enhancing plasminogen activation [16]. Differentiation medium was supplemented with EGCG, and the TN protein secreted into the medium was assessed following the myogenic differentiation of C2C12 cells (Figure 6). Western blot analysis showed that TN levels in the medium decreased with nearly complete depletion by day 6 of differentiation (Figure 6A,B). In contrast, the control without EGCG supplementation showed an increase in medium TN levels. Moreover, TN depletion in the medium was dose-dependent on EGCG, indicating that EGCG could effectively remove TN from the differentiation medium (Figure 6C,D). Furthermore, TN gene expression was also significantly downregulated by EGCG in a dose-dependent manner (Figure 6E).

### 3.5. EGCG Promotes Myogenic Differentiation via the Inhibition of TN

**Figure 7:**
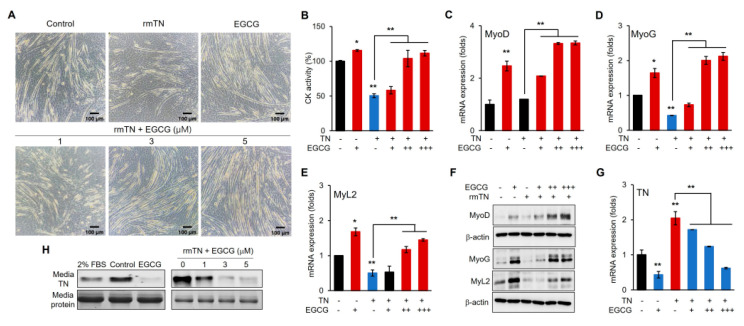
EGCG-promoted myogenic differentiation mediated by inhibiting TN. (**A**) Comparison of microscopic figures (bright field) on day 6 of differentiation with indicated supplementations (rmTN = 2 μM). (**B**) Comparison of creatine kinase activity (CK). (**C**–**E**) mRNA expression of myogenic marker genes. (**F**) Immunoblotting for indicated marker proteins on day 6 of differentiation with indicated supplementations. (**G**) mRNA expression of myogenic marker genes and TN gene. Values from *n* ≥ 3 with standard deviations and *p* values ≤ 0.05 (*) or ≤0.01 (**). (**H**) Immunoblotting for TN in media from (**A**).

The myogenic differentiation of C2C12 cells was further assessed with increasing concentrations of EGCG in the presence of a fixed concentration of exogenous TN (Figure 7). EGCG dose-dependently reversed the TN-induced suppression of differentiation and promoted myogenic differentiation, as evidenced by increased myotube formation and CK activity (Figure 7A,B). EGCG dose-dependently upregulated the marker genes MyoD, MyoG, and MyL2 (Figure 7C–E), accompanied by corresponding increases in protein levels (Figure 7F). Moreover, EGCG dose-dependently downregulated TN gene expression, while exogenous TN upregulated TN gene expression (Figure 7G). The analysis of TN protein levels indicated that EGCG dose-dependently depleted endogenous TN and removed exogenously supplemented TN (Figure 7H).

## 4. Discussion

Previous studies have suggested that TN is involved in regulating various types of stem cell differentiation, including myogenesis. The significant upregulation of TN during embryonic skeletal muscle development and muscle stem differentiation has been reported [8,18]. Moreover, recent studies have identified TN as a potential diagnostic marker of heart failure and reported its association with ischemic heart disease [19,20]. However, the functional role of TN remains poorly understood. In this study, we demonstrated that tetranectin (TN) is significantly upregulated and secreted during the myogenic differentiation of C2C12 satellite cells. The secretion of TN suggests its extracellular role in the differentiation process. Importantly, exogenous TN supplementation suppressed myogenic differentiation (Figure 2). In addition, the depletion of TN in the differentiation medium enhanced differentiation (Figure 3), indicating that TN acts as an anti-myogenic factor, negatively regulating myogenic differentiation. These results also implicate the multifunctional roles of TN in extracellular matrix remodeling and cell signaling, although its regulatory mechanism remains to be elucidated.

The major green tea polyphenol EGCG has been shown to exert various biological effects, including the modulation of cell proliferation and differentiation. In our study, low concentrations of EGCG enhanced C2C12 myogenic differentiation without affecting cell proliferation (Figure 4 and Figure 5), which is consistent with reports that polyphenols can positively regulate muscle regeneration [12,13,14]. Mechanistically, we demonstrated that EGCG effectively removes TN proteins from the differentiation medium and downregulates TN gene expression in a dose-dependent manner (Figure 6). Moreover, EGCG reversed the TN-induced suppression of myogenic differentiation, restoring the expression of myogenic markers and enhancing protein levels (Figure 7). This indicates that EGCG promotes myogenesis at least in part by inhibiting TN, and indicates the molecular interaction between EGCG and TN, which is consistent with previous reports [16]. Interestingly, exogenous TN supplementation increased TN gene expression, indicating an autologous activation mechanism (Figure 7G). This upregulation downregulated EGCG dose-dependently, likely by removing extracellular TN and thereby disrupting this autologous feedback loop (Figure 6E and Figure 7G). These results strongly suggest that EGCG promotes myogenic differentiation by both inhibiting the extracellular activity of TN and downregulating its gene expression.

Although the exact mechanisms by which TN negatively regulates myogenic differentiation remain to be fully elucidated, the results in this study suggest that TN primarily functions in the extracellular compartment. TN was predominantly detected in the differentiation medium, and its levels increased following myogenic differentiation (Figure 1 and Figure 6). In contrast, intracellular TN was undetectable, despite the significant upregulation of TN gene expression. Similarly, exogenously supplemented TN remained mostly in the medium, and intracellular transport of TN was negligible (Figure 7). Previous studies have reported the presence of TN in muscle cells during the early stage of mouse embryonic development [8]. However, in later developmental stages, TN accumulates at myotendinous junctions. Moreover, in differentiated C2C12 cells, TN is primarily detected on the surface of myotubes [8]. These observations, together with our results, suggest that TN may localize to distinct cellular compartments at different stages during myogenic differentiation. We further speculate that TN functions extracellularly, binding to a receptor or a mediating protein, thereby initiating the anti-myogenic signaling pathway.

In conclusion, we identify TN as an anti-myogenic factor that is upregulated and secreted during C2C12 differentiation. Although the regulatory mechanism of TN in the suppression of myogenesis remains to be fully elucidated, this study expands our understanding of the regulatory network controlling myogenic differentiation and highlights TN as a potential therapeutic target for muscle regeneration. Furthermore, we demonstrate that EGCG promotes myogenesis by inhibiting TN function and expression. These findings suggest that targeting TN with compounds like EGCG could be a promising strategy for the treatment of muscle-related disorders characterized by impaired differentiation or regeneration.

## Data Availability

The datasets generated and/or analyzed during the current study are available from the corresponding author on reasonable request.

## Supplementary Materials

The following supporting information can be downloaded at https://www.mdpi.com/article/10.3390/cells14151160/s1, Table S1: Primers for RT-PCR; Table S2: Antibody for Western blot analysis.

## Abbreviations

The following abbreviations are used in this manuscript:

TN	Tetranectin
CK	Creatine kinase
EGCG	Epigallocatechin-3-gallate
MyoD	Myoblast determination protein 1
MyoG	Myogenin
MyL2	Myosin regulatory light chain 2
Plg	Plasminogen
TN-del-FBS	Tetranectin-depleted fetal bovine serum
