# Supplementary material for: The Anti-Myogenic Role of Tetranectin and Its Inhibition by Epigallocatechin-3-Gallate Enhances Myogenesis
Source: Cells. 2025 Jul 28;14(15):1160. doi: 10.3390/cells14151160 (PMC12346571; doi:10.3390/cells14151160)
Supplement: Supplementary file 1 [file cells-14-01160-s001.zip › cells-3775480-supplementary.pdf]

Supplementary Table S1. Primers for RT-PCR.

Gene	Sequence 5'-3'
β-Actin	Forward: ACCCTAAGGCCAACCGTG<br>Reverse: GCCTGGATGGCTACGTAC
TN	Forward: CTGAAGGGCACCAAGGTGAA<br>Reverse: TCCCAGTTCTTGTAGGCCAG
MyoD	Forward: AGGAGCACGCACACTTCTCT<br>Reverse: TCTCGAAGGCCTCATTCACT
Myog	Forward: TCCAGTACATTGAGCGCCTA<br>Reverse: CAAATGATCTCCTGGGTTGG
MyL2	Forward: AAAGAGGCTCCAGGTCCAAT<br>Reverse: CCTCTCTGCTTGTGTTGGTCA

Spplementary Table S2. Antibody for western blot analysis.

Antibody	Providers	Catalog #
Anti-TN	Abcam	Ab108999
Anti-MyoD	Thermo Scientific	MA5-12902
Anti-MyoG	Santa Cruz	Sc-12732
Anti-MyL2	Abcam	Ab48003
